# Whisky Hypodermically in Collapse from Loss of Blood

**Published:** 1879-05-20

**Authors:** W. N. Ames

**Affiliations:** Miss.


### *WHISKEY HYPODERMICALLY IN COLLAPSE FROM LOSS OF BLOOD.*

BY. W. N. AMES, M.D., OF MISS.

Mrs. H—— aborted at the third month, and sent for me to take away the placenta. On arrival I found her almost exsanguined from loss of blood, with the placenta adherent. I made a vigorous effort to take away the placenta by introducing my right hand into the vagina and making supra-pubic pressure with my left, in order to steady the uterus and press it lower into the pelvis. My index finger touched the placenta, but could not introduce it far enough to tear it loose. My patient was almost pulseless; respiration sighing, and her pupils enormously dilated. Surface of her body suffused with cold perspiration. As soon as I had discovered that there must necessarily be some delay in removing the placenta, I used the tampon and gave the patient whiskey toddy, which she vomited instantly. I then injected under the skin whiskey and water, in which was dissolved some ergotine. During the treatment of about four hours, twenty grains of ergotine were used. The injections of whiskey were repeated as often as possible until six syringefuls were used. Care was taken in the meantime to lower the head of the bed, by propping the foot. She fainted several times, even while her head was lower than her body.

The loss of blood was enormous. The hypodermic injections of whiskey were repeated perhaps twenty times before the pulse could be detected at the wrist, and after a lapse of four hours. Her stomach was irritable as from profound shock from severe injury.

The above treatment was suggested from reading a paper in the Transactions of the Mississippi State Medical Association, by the late Dr. Whitehead, of Vicksburg, on the use of verat. viride hypodermically in the treatment of puerperal convulsions. Dr. Whitehead controlled the convulsions by the veratrum, and produced complete prostration, and in order to antidote the remedy, he successfully injected whiskey. The injections were made in the forearm with only one abscess resulting.

In twenty-four hours the tampon was removed, with which the placenta came away. The woman made a slow recovery. The whiskey had no other effect than to relieve the collapse, and that very slowly.

